# Occlusal equilibration and muscle activity in fixed versus removable mandibular implant supported overdenture

**DOI:** 10.1007/s00784-024-05558-w

**Published:** 2024-02-21

**Authors:** Asmaa N. Elboraey, Wessam M. Dehis, Sherihan M. Eissa, Menatallah Mohamed El-Hotieby, Omar K. Abdelkader

**Affiliations:** 1 Fixed and Removable Prosthodontics Dep. Oral and Dental Research institute, National Research Centre, Cairo, 12622 Egypt; 2 Researcher Fixed and Removable Prosthodontics Dep. Oral and Dental Research institute, National Research Centre, Cairo, 12622 Egypt

**Keywords:** Occlusal Equilibration, Muscle activity, Removable overdenture, Fixed prostheses, Implant supported prosthesis, Mandibular implant-supported overdenture

## Abstract

**Objectives:**

Single denture rehabilitated patients have negative appraisals regarding oral function, mostly associated by stability and retention issues regarding mandibular prosthetics. Therefore, this study assessed patients’ occlusal equilibration, muscle activity, and oral health-related quality of life (OHRQoL) receiving milled removable or fixed mandibular implant retained prostheses.

**Materials and methods:**

Twenty-two edentulous mandibular ridges patients were randomly distributed into two groups based on the definitive prosthesis received. Group I: Removable mandibular implant-supported overdenture, Group II: Implant retained fixed prosthesis. Occlusal equilibration was evaluated utilizing Occlusense, muscle activity via Electromyograph (EMG) at delivery, after one, and three months. The OHRQoL was evaluated by Oral Health Impact Profile questionnaire (OHIP-19) before delivery and after follow-ups. Data were collected, tabulated, and analyzed, utilizing independent t-test and One-way ANOVA followed Tukey`s post-hoc test. Significance level set at *P* ≤ 0.05.

**Results:**

Groups I &II showed significant improvement in occlusal equilibration, muscle activity and OHRQoL. Group II demonstrated significantly higher improvement than group I in occlusal equilibration associated with muscle activity after 1 month, and in functional limitations domain in OHRQoL questionnaire after 3 months.

**Conclusion:**

Implant retained mandibular prosthesis showed improvement in occlusal equilibration, muscle activity, and OHRQoL regardless of prosthesis type employed. Fixed implant-supported prosthesis revealed better outcomes than removable mandibular implant-supported overdenture concerning occlusal equilibration, muscle activity, and OHRQoL regarding functional limitations.

**Clinical relevance:**

Implant retained mandibular prosthesis is one of best treatment options for single mandibular completely edentulous patients, as dental implants improved occlusal equilibration, muscle activity, and OHRQoL.

## Introduction

One of the most advanced treatment modalities recommended for patients with single mandibular edentulous ridge is Computer-aided design and computer-aided manufacture (CAD/CAM) milled implant retained prostheses either fixed or removable. These restorations are considered a life-changing treatment resolving edentulism, upgrading prostheses retention and stability, and consequentially providing superior quality of life [1].

The main goal of implant-supported prostheses is the long-term stability of all components of the masticatory system. Occlusal equilibration is one of the key factors for maintaining the harmony of the stomatognathic system. Optimum occlusal equilibration is considered a highly essential challenge facing implant-supported prostheses either fixed or removable in mandibular arches. Its importance is accredited to its great impact on prosthesis retention, support, and stability together with the remarkable diminishing of unbalanced forces, there are several methods of occlusal equilibration evaluation referring to articulating papers as an example [2].

Rapid advancements in technology have occurred in this modern digital era allowing the opportunity of using advanced devices to evaluate and analyze occlusion such as Occlusense. Occlusense is an objective assessment tool used to evaluate the occlusion of a patient. Unlike articulating papers, which can only determine location, it can identify both force and timing; two of the most fundamental parameters for measuring occlusion. The device sensors are flexible, pressure-sensitive transducers embedded in an intraoral recording wireless handpiece connected to the recording device. This recent version displays two- and three-dimensional graphical analysis and records the occlusal contact forces distribution and symmetry preliminary starting with initial contact till maximum intercuspation [3, 4].

Occlusal equilibration defined as modification of the occlusal form of the teeth with the intent of equalizing occlusal stress, producing simultaneous occlusal contacts or harmonizing cuspal relations and it is one of the key factors to have optimum muscular activity [5]. Muscle activity is an essential parameter in assessing prosthesis success via contributing to swallowing procedure, digestion activity, nutrition status, and successively patient’s general health. Surface electromyography (EMG) is one of the most common and accurate methods to evaluate muscular activity. Muscular activity is mandatory in sustaining the integrity of the stomato-gnathic system to fragment, mash, and grind food [5–7]. Mandibular edentulous patients not only seek function but also aesthetics, self-confidence, welfare, and superior quality of life. Accordingly, estimation of any probable discomfort, dysfunction, and disability in rehabilitated edentulous patients and geriatric populations is essential via utilizing the OHIP (Oral Health Impact Profile) which comprises lengthy forty-nine objects, founded on seven theoretical models which are not clinically practical. Consequently, a shorter form that comprises only nineteen questions attained from the original OHIP was established and identified as (OHIP-19) [8, 9].

Even though CAD/CAM mandibular implant-retained prostheses whether fixed or removable are highly advantageous, it has been reported that edentulous patient’s occlusal equilibration, masticatory performance, and health-related quality of life vary depending on the prosthesis type utilized in restoring such patients. Accordingly, this investigation assessed occlusal equilibration, muscle activity, and oral health-related quality of life (OHRQoL) related to patients treated by fixed versus removable mandibular implant-retained prostheses. The null hypothesis was no variance in these parameters.

### Patients and methods

#### Study design

Twenty-two patients with single edentulous mandibles aged 55–60 years were chosen from the outpatient dental clinic of the Medical and Scientific Centre of Excellent (MSCE) of the National Research Centre (NRC) Cairo, Egypt. Each patient’s mandible received six dental implants. Patients were randomly distributed with 1:1 allocation ratio into two groups based upon the final restoration type.

Group I obtained a removable mandibular implant-supported overdenture. Group II obtained implant-retained fixed prosthesis. Occlusal equilibration and muscle activity were assessed on delivery, after one and three months. While the OHRQoL was assessed before delivery and at the end of follow up period.

#### Ethical approval and clinical trial registration

The current study has been applied with the Code of Ethics of the World Medical Association, following the ethics stated in the Declaration of Helsinki in 1975. This research has been permitted by the Medical Research Ethical Committee of the National Research Centre, Cairo, Egypt with agreement number 14,312,012,023. All patients were up-to-date with the existing study’s practical phases and contracted with printed consent. The study was registered on clinicalTrials.gov under the identifier: NCT05949151.

#### Sample size calculation

The Sample size was estimated depending on a previous study by Alloush et al. [10] as a reference. Accordingly, when the mean ± standard deviation of masseter muscle activity after 1 month of implant insertion in implant-retained fixed overdenture was (316.1 ± 87.5), while the estimated mean of the other group was 190, the minimally accepted sample size was 9 patients per group when the power was 80% and type I error probability was 0.05. The total sample size was enlarged to 11 patients per group to compensate for a 20% dropout. The independent t-test was employed to calculate sample size by using G. power version 3.1.9.7.

#### Inclusion and exclusion criteria

The inclusion criteria were male patients with a single edentulous mandible (having opposing natural dentition) with a well-developed alveolar ridge, free from temporomandibular joint disorder and any chronic disease. Though exclusion criteria were smokers, patients having systemically immunosuppressive diseases, or radiotherapy-treated ones.

## Methods

### Implant insertion

Each patient was radiographically evaluated using cone beam computed tomography (CBCT) (CAT 17–19, Imaging Sciences International, Hatfield, PA). The accurate site and size of the dental implants were determined by a software program (Bluesky Bio) which allows the fabrication of surgical guides that provide accurate virtual installation of the six dental fixtures (K1 line conical connection double thread, OXY, Italy) varied from 4 to 5 mm in width and 7 to 10 mm in length according to each site based on bone dimensions and they were virtually located in the mandible utilizing the flapless technique.

Implants stayed unloaded for three months to ensure optimum osseointegration. Patients received a conventional mandibular complete acrylic denture and were recalled for frequent follow-up visits within the 3 months of osseointegration. Afterward, the final prosthetic stage took place and patients were randomly split into two equivalent groups as stated by the type of definitive restoration received. Group (I): mandibular removable overdentures supported and retained by ball and socket attachments. Group (II): mandibular implant-retained fixed prosthesis.

### Removable and fixed prostheses construction

The primary impression was recorded utilizing irreversible hydrocolloid impression material and transferred into a stone cast. The custom acrylic open tray was fabricated. The implant level transfer copings were screwed to the implant fixtures and immobilized with Duralay acrylic resin (Duralay, Reliance Dental MFG Co, Worth, IL, USA) avoiding transfer coping mobility thru the impression process. Light and heavy body rubber base impressions (SPEEDEX, Coltene/Whaledent Pvt., Ltd., 9450 Alstalten, Switzerland) were employed for secondary impression recording the accurate position of the transfer copings. Implants’ analogs were attached to transfer copings where the master cast was obtained following impression pouring. Record blocks were made-up, the jaw relationship was recorded, and appropriate lip support was reestablished.

On the master cast the transfer copings were detached and ball abutments were screwed into the implant analogs. A block-out shim was modified for each abutment for blocking out the implant’s undercuts. Then the cast with the ball abutments and housing caps was sprayed with Opti scan spray (SIRONA CEREC Optispray, Germany) and scanned with a LAB scanner (Ceramill Map400, Amann Girrbach AG. Koblach, Austria). The digital overdentures’ designs (Exocad Dental CAD, GmbH) were milled (Ivotion base and tooth discs, Ivoclar Vivadent, Schaan, Liechtenstein) with relief areas against the prospective site of titanium housing caps.

On delivery the ball abutments were screwed to the dental implants with proper torque according to the manufacturer’s instructions and housing caps were attached to them, the implant’s undercuts were blocked out by pink wax, and the overdenture was checked for any required additional relief to accommodate the Duralay resin for the housings pick-up. The denture was completely seated, and the patient was coached to gently bite on it throughout the Duralay setting period. Subsequently, the denture was detached and the housings within the denture were inspected, then the overdenture occlusion was checked, and any further adjustments needed were accomplished.

Regarding the fixed prostheses (bridges) of group II, the implants’ abutments were screwed to the implants’ analogs in the master cast. Thereafter the master cast was sprayed with Opti scan spray and scanned with a LAB scanner. The fixed prosthesis was designed with the aid of CAD software into one full arch bridge. The prosthesis was milled twice; incipiently with Polymethyl methacrylate (PMMA) for try-in thenceforth with monolithic Zirconia (Cercone, 25 mm, Dentsply Sirona), and pink porcelain was utilized at the gingival areas for esthetic considerations. Occlusion was checked and any necessary adjustments were carried out.

Occlusal equilibration and muscle activity were assessed once the prosthesis was delivered, post one and three months. While the OHRQoL was assessed prior to denture insertion and post follow-up period termination.

### Assessment of occlusal equilibration

Occlusal equilibrations were estimated by Occlusense (Bausch GmbH & Co. KG) and the proper sensor size was both chosen for each patient and attached to the Occlusense handpiece which was then linked to an Apple I pad thru Bluetooth technology. The sensor was inserted intra-orally and positioned parallel to the occlusal plane as much as possible. Then the patient was requested to bite, and the occlusal contacts were detected on the I Pad screen. This procedure was repeated 3 times at each assessment appointment and all the records were stored in the I Pad.

### Assessment of muscle activity

EMG recordings were accomplished by a computer electromyography-based data acquisition system (Deymed TRU- TRACE EMG NCV 4 channel System machine (AU7-12060002) present at Rheumatology and Nerve conduction and Electromyography clinic of MSCE of NRC. Average quantities and sizes of both cucumber and nuts were chewed, till they were ready for swallowing, to minimize patient unpredictability. Throughout all records, the patients were requested to sit in an erect natural posture while the head was unsupported. A silver chloride/silver metal surface bipolar electrode (active) was fixated in the middle of the superficial belly of the masseter then temporalis along with the direction of the fibers’ long axis, while the other electrode (reference) was fixated to the chin of the patient. The skin was disinfected with 70% alcohol before electrode placement to lessen resistance, then an electro-conductive gel was spread on the electrodes prior to skin contact and they were secured by adhesive tapes.

Then patients were instructed to chew on their prosthesis inducing facial muscle contraction creating impulses that were laser printed. One second sweeping speed and 200 µV sensitivity were adjusted. Amplification, smoothening, and filtering at 20 Hz − 10 kHz of signals were executed. The procedure was repeated several, fixed number of times for each type of food, with both muscles, and 2 min window of recovery followed by mean analysis. Single, blinded, and experienced practitioner performed the test, acquired the data, and analyzed them utilizing the device software.

### Assessment of OHRQoL

OHRQoL was estimated by employing the OHIP-19 which comprises nineteen multiple questions that were translated into Arabic. It consists of seven segments: functional limitation, handicap, both physical disability and pain, together with psychological discomfort, accompanying both social and psychological disability. Each multiple-choice question has five answers to choose from, where every choice has a definite score (4 = very often, 3 = often, 2 = fairly often, 1 = seldom, 0 = never) ranging between 0 and 76. The total score was estimated by gathering replies to all questions. The least score revealed a satisfactory sense of the patient’s oral condition and consequently superior satisfaction, pleasure, and quality of life (10).

### Statistical analysis

All numerical data were presented as mean and standard deviation. Normality exploration of the given data was completed utilizing Shapiro-Wilk and Kolmogorov-Smirnov tests for normality which revealed that all data originated from normal distribution as *P*-value > 0.05 regarding occlusal equilibration and EMG calibrations. Accordingly, differentiation between both groups was achieved by employing Independent T-test, while assessment among dissimilar intervals was applied using the One-Way ANOVA test accompanied by Tukey`s Post Hoc test for multiple comparisons. On the other side, normality exploration revealed significant differences (non-parametric data) regarding OHIP scores. Hence, comparison among different groups was carried out utilizing Mann Whitney’s test, while differentiation between before and after was performed using Wilcoxon Signed Rank. The significance level was set at the level of *P* ≤ 0.05.

## Results

### Assessment of occlusal equilibration

**Fig. 1:**
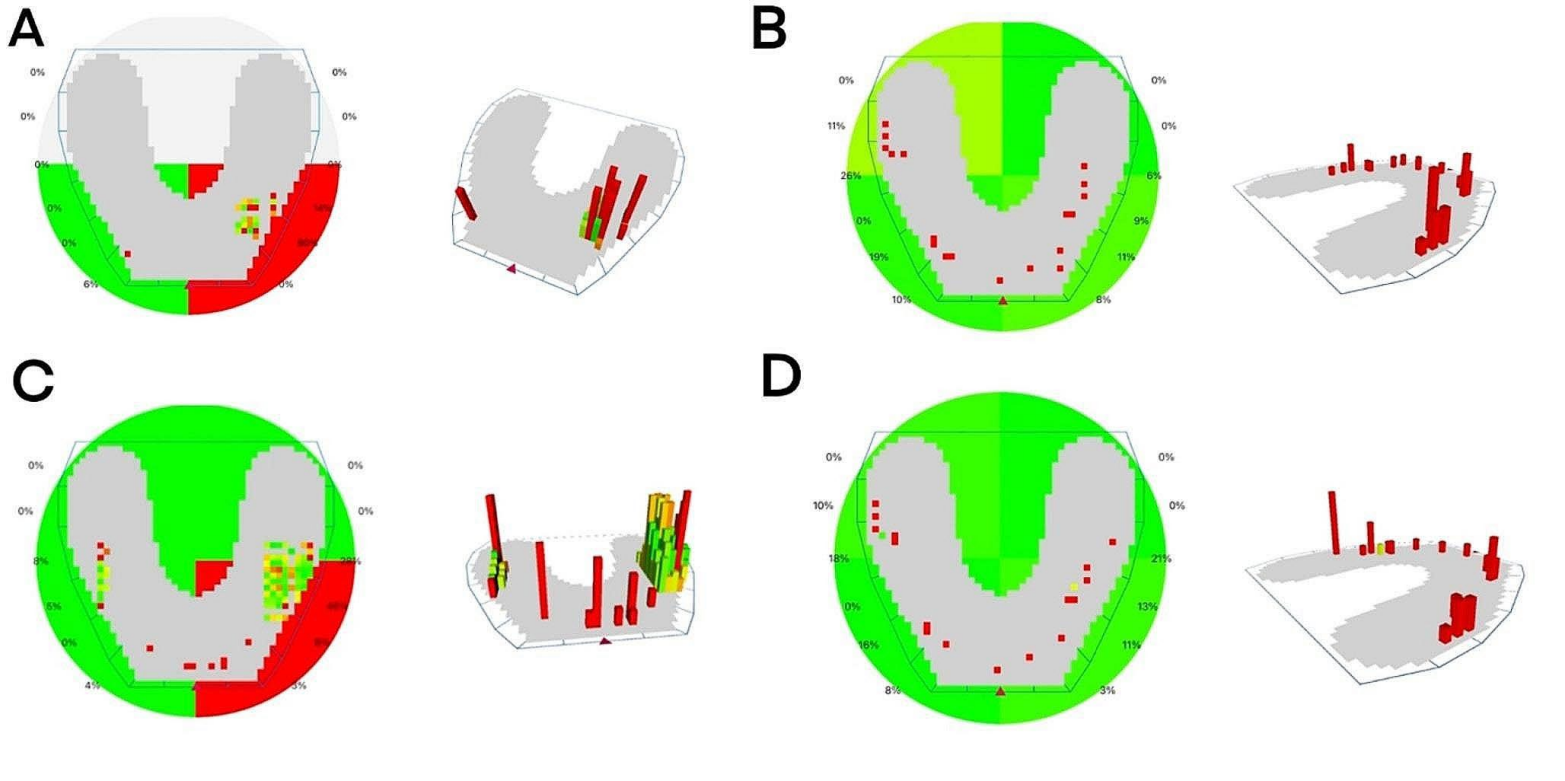
(**A**) Occlusal forces distribution in removable overdenture at delivery and (**B**) at the end of follow-up. (**C**) occlusal forces distribution in fixed prosthesis at delivery and (**D**) at the end of follow-up

Comparison among follow-up intervals to estimate the time impact revealed a significant decline of occlusal force at right and left anterior areas in both groups, while such force increased remarkably over time at right and left posterior areas in both groups as *P* < 0.05 (Table 1). Evaluating the prosthesis type effect in both groups displayed that; at delivery, there was an insignificant difference in occlusal force distribution between both groups as *P* > 0.05 concerning right, left, anterior, and posterior areas. After 1 and 3 months; group II had remarkably lower anterior occlusal force and higher posterior occlusal force than group I as a *P* value < 0.05 (Table 2; Fig. 1). Assessment of all areas in each group implied that the right and left anterior force was significantly lower than the posterior one at all intervals.

**Table 1:** Effect of follow-up intervals on occlusal forces distribution at different areas in both groups

Area		Side	At delivery		After 1month		After 3months		*P* value
			M	SD	M	SD	M	SD	
Group I	Anterior area	Right	17.53 ^a^	2.80	12.32 ^b^	1.48	11.18 ^b^	1.34	< 0.0001*
		left	18.90 ^a^	3.02	13.87 ^b^	1.66	10.2 ^c^	1.22	0.005*
	Posterior area	Right	32.10 ^a^	5.14	34.92 ^ab^	4.19	39.12 ^b^	4.69	0.005*
		left	31.24 ^a^	4.99	38.27 ^b^	4.59	40.01 ^b^	4.8	0.0004*
Group II	Anterior area	Right	17.50 ^a^	2.80	7.68 ^b^	1.38	6.15 ^b^	1.01	< 0.0001*
		left	16.90 ^a^	2.70	6.93 ^b^	1.25	5.89 ^b^	0.97	< 0.0001*
	Posterior area	Right	33.10 ^a^	5.30	41.68 ^b^	7.5	43.85 ^b^	7.23	0.001*
		left	32.50 ^a^	5.00	42.87 ^b^	7.72	44.32 ^b^	5.15	0.001*

M: mean SD: standard deviation *Significant difference as *P* < 0.05

Means with different superscript letters per raw were significantly different as *P* < 0.05

Means with the same superscript letters per raw were insignificantly different as *P* > 0.05

**Table 2:** Effect of prothesis type on occlusal forces distribution at different areas at different follow-up intervals

Interval	Area	Side	Group I		Group II		Difference (Independent t test)				
							MD	SEM	95% CI		*P* value
			M	SD	M	SD			L	U	
At delivery	Anterior area	Right	17.53 ^a^	2.80	17.5 ^a^	2.80	0.42	1.06	-2.60	1.80	0.69
		left	18.9 ^a^	3.02	16.9 ^a^	2.70	2.00	1.22	-0.55	4.50	0.11
	Posterior area	Right	32.1 ^b^	5.14	33.1 ^b^	5.30	1.00	2.22	-5.64	3.64	0.66
		left	31.24 ^b^	4.99	32.5 ^b^	5.00	1.26	2.13	-5.71	3.18	0.56
	*P* value (One Way ANOVA)		< 0.0001*		< 0.0001*						
After 1 month	Anterior area	Right	12.32 ^a^	1.48	7.68 ^a^	1.38	4.64	0.61	3.36	5.91	< 0.0001*
		left	13.87 ^a^	1.66	6.93 ^a^	1.25	6.94	0.62	5.63	8.24	< 0.0001*
	Posterior area	Right	34.92 ^b^	4.19	41.68 ^b^	7.50	6.76	2.59	-12.16	-1.35	0.016*
		left	38.27 ^b^	4.59	42.87 ^b^	5.11	4.61	2.06	-8.91	-0.28	0.03*
	*P* value (One Way ANOVA)		< 0.0001*		< 0.0001*						
After 3 months	Anterior area	Right	11.18 ^a^	1.34	6.15 ^a^	1.01	5.03	0.51	3.97	6.08	< 0.0001*
		left	10.2 ^a^	1.22	5.89 ^a^	0.97	4.31	0.46	3.33	5.29	< 0.0001*
	Posterior area	Right	39.12 ^b^	4.69	43.85 ^b^	5.26	4.73	2.12	-9.16	-0.29	0.03*
		left	40.01 ^b^	4.80	44.32 ^b^	5.15	4.31	1.98	-8.45	-0.16	0.04*
	*P* value (One Way ANOVA)		< 0.0001*		< 0.0001*						

M: mean SD: standard deviation *Significant difference as *P* < 0.05

Means with different superscript letters per column were significantly different as *P* < 0.05

Means with the same superscript letters per column were insignificantly different as *P* > 0.05

MD: mean difference SEM: standard error mean CI: confidence interval L: lower arm U: upper arm

### Assessment of muscle activity

Estimating the influence of time by comparing muscle activity at various intervals declared a significant increase in both groups as *P* < 0.05 (Table 3). Group, I showed a remarkable improvement in temporalis and masseter muscle activity after 3 months, though an insignificant difference was between delivery and post-1-month. On the other hand, group II revealed a significant improvement in temporalis and masseter muscle activity after 1-month and significantly sustained till the end of follow-up. Differentiation between both groups for determining prosthesis’s type influence displayed that at delivery there was an insignificant variance between both groups as *P* value > 0.05 concerning temporalis and masseter muscles. After 1- and 3 months group II revealed remarkably higher muscular activity than group I as *P* value < 0.05.

**Table 3:** Evaluation of temporalis muscle and masseter muscle activity (µv) at different follow-up intervals in both groups

Muscle	Group	At delivery		After 1 month		After 3 months		*P* value
		M	SD	M	SD	M	SD	
Temporalis	Group I	151.3 ^a^	33.286	168.7 ^a^	37.114	191.3 ^b^	42.086	0.01*
	Group II	150.9 ^a^	33.198	230.8 ^b^	50.776	278.3 ^b^	61.226	0.0001*
	*P* value	0.97		0.004*		0.001*		
Masseter	Group I	179.4 ^a^	39.468	210.8 ^a^	46.376	248.9 ^b^	54.758	0.007*
	Group II	175.5 ^a^	38.61	331.8 ^b^	72.996	421.6 ^b^	92.752	0.0001*
	*P* value	0.81		0.0001*		0.0001*		

M: mean SD: standard *Significant difference as *P* < 0.05

Mean with the same superscript letters were insignificantly different as *P* > 0.05

Means with different superscript letters were significantly different as *P* < 0.05

### Assessment of OHRQoL

Comparison between pre- and post-prosthesis insertion to estimate the influence of time revealed a significant decline in scores (improvement in OHIP-19) as a *P* value < 0.05 regarding both groups (Table 4). Whereas differentiation among both groups to estimate the effect of prosthesis type revealed insignificant differences between them at all sections and total OHIP-19 as *P* value > 0.05, except in the functional limitation segment; where group II demonstrated significantly lower score compared to group I.

**Table 4:** OHIP-19 score before and after 3 months in both groups

OHIP	Group	Before delivery		After 3 months		*P* value
		M	SD	M	SD	
Functional limitation	Group I	9.54	0.82	2.18	0.75	0.003*
	Group II	9.55	0.82	1.54	0.52	0.003*
	*P* value	0.99		0.01*		
Physical pain	Group I	12.82	1.08	2.19	0.61	0.003*
	Group II	12.27	1.68	1.81	0.40	0.003*
	*P* value	0.51		0.21		
Psychological discomfort	Group I	6.18	0.75	2.11	0.72	0.003*
	Group II	6.27	1.62	2.09	0.70	0.003*
	*P* value	0.84		0.99		
Physical disability	Group I	10.36	0.67	1.73	0.65	0.003*
	Group II	10.09	0.94	1.82	0.75	0.003*
	*P* value	0.56		0.84		
Psychological disability	Group I	5.45	0.52	1.45	0.52	0.002*
	Group II	5.18	0.87	1.64	0.67	0.003*
	*P* value	0.56		0.61		
Social disability	Group I	8.64	0.92	1.73	0.65	0.003*
	Group II	8.55	0.52	1.81	0.75	0.003*
	*P* value	0.84		0.84		
Handicap	Group I	6.27	0.79	2.09	0.71	0.003*
	Group II	6.18	0.60	2.08	0.83	0.004*
	*P* value	0.69		0.99		
Total OHIP-19	Group I	59.27	1.85	13.45	1.69	0.003*
	Group II	58.09	2.07	12.82	1.17	0.003*
	*P* value	0.24		0.43		

M: mean SD: standard deviation MD: mean difference SED: standard error difference

CI: confidence interval L: lower arm U: upper arm

*Significant difference as *P* < 0.05

## Discussion

The available prosthetic restorative options for rehabilitation of completely edentulous patients regarding final restoration were either removable or fixed especially after implant placement; each option had its own merits and demerits. With the development of new technologies and equipment, researchers have the opportunity to assess the most successful treatment option. Therefore, this study was designed to assess occlusal equilibration, muscle activity, and OHRQoL of fixed and removable implant retained prosthetics restoring single edentulous mandible [11–14].

Occlusense is a reliable intraoral computerized diagnostic device for achieving occlusal equilibration, offering superior accuracy, sensibility, and stability, detecting premature contact areas, divergences, and homogenously dispersed occlusion [15, 16]. In this study, occlusal equilibration in both groups was achieved following multiple occlusal adjustments before prosthesis delivery. Moreover, initial non-uniform occlusal force distribution showed high anterior force that decreased over time, while posterior force increased due to patients’ adaptation and harmony. Periodically force rose posteriorly rather than anteriorly may be related to post-prosthetic adaptability and proper fit in both types of prostheses [17].

Better force distribution was gained by time in removable mandibular implant-supported overdenture by accomplishing bilaterally balanced occlusion avoiding denture dislodgments, whereas implant-retained fixed prostheses showed better force distribution. This result is in accordance with another study that delineated that the cement layer has a cushioning effect that dissipates stresses at the abutment-implant interface and provides more controlled force direction and analysis [18].

EMG muscle activity assessment is crucial for evaluating patients’ functional masticatory performance after oral rehabilitation. Increased muscle activity by time in implant-supported prostheses may be due to improvement of masticatory efficiency, neuro-muscular adaptation, stability, support, and retention of the prosthetics, oral perception enhancement, obtaining occlusal plane stability, and improved patient’s comfort in chewing process in comparison with conventional dentures. Moreover, it was reported that this improvement may be due to osseo-perception, improving tactile and stereognosis abilities, and periosteal mechanoreceptors giving a sensory reaction rather than periodontal ligament. Other studies related it to the vast positive influence and impact of implant treatment on patient satisfaction, maximum intercuspation force, and muscular activity [1, 19].

At prosthesis insertion, there was insignificant variance among both groups regarding masticatory performance. This simply clarified that regardless of the prosthesis form employed, initially, the patient’s mastication and neuromuscular adaptation are poor and are habitually enhanced steadily. Subsequently, the development of favorable masticatory performance took place in both groups, however, group II was superior to group I in this study. Such a finding coincides with other investigations mentioning the fact that the degree of muscle activity mainly relies on the nature of prosthetic support, stability, and retention [20–22].

The study demonstrated that implant-supported prostheses significantly improved OHRQoL in edentulous patients. Improvement was achieved through reduced functional complaints, improved appearance, and reduced functional limitations. The implant-supported prosthesis also reduced inappropriate jaw alignment, physical, and psychological disabilities [20, 23].

These research findings disclosed a non-remarkable difference among both groups though group II interpretations are insignificantly better than group I concerning psychological discomfort, psychological disability, physical pain, physical disability, social disability, and handicap, besides total OHIP-19 that may be due to utilization of, dental implants supporting and fixating the overdenture by ball and socket attachments, giving the of sensation nearly fixed prosthesis [24].

On the other hand, there was a remarkable variation between both groups concerning functional limitation, as implant-supported overdenture has less chewing ability, more bulk, and maximum contact with the underlying mucosa compared with fixed prostheses. Nevertheless, fixed prostheses apply higher bite forces, and increase the efficiency of grinding foods during the masticatory process. Therefore, overdentures wearers have nutritional restrictions that affect the functional limitation domain in the OHRQoL questionnaire. In agreement with this study, it was demonstrated that removable prostheses showed lower quality of life if compared with fixed which could have been related to prostheses’ clinical features, regarding lateral movements and rotation, mucosal contact, lesser maximum occlusal force, and more maintenance visits which were nearly kept to the minimum in this study having a minor influence because of the abundant number of implants used giving more favorable response [25, 26].

### Limitations and recommendations

The short-range follow-up period was the limitation faced during the study. Accordingly, upcoming crossover research with prolonged follow-up intervals to accurately record the perception of the same patient to different types of prosthetics used in rehabilitation is suggested to take place by calibrating the same parameters in patients with a single edentulous mandibular ridge.

## Conclusion

Based on the findings of the presented research, the forthcoming conclusions were attained; Implant retained mandibular prosthesis is considered a beneficial line of treatment for single mandibular completely edentulous patients, it showed improvement in occlusal equilibration, muscle activity, and OHRQoL regardless of prosthesis type employed. Fixed implant-supported prosthesis revealed better outcomes than removable mandibular implant-supported overdenture concerning occlusal equilibration, muscle activity, and OHRQoL regarding functional limitations.

## Abbreviations

OHRQoL: Oral health-related quality of life
EMG: Electromyograph
OHIP-19: Oral Health Impact Profile questionnaire
CAD/CAM: Computer-aided design and computer-aided manufacture
NRC: National Research Centre
CBCT: Cone beam computed tomography
PMMA: Polymethyl methacrylate
MSCE: Medical and Scientific Centre of Excellent
